# Risk factors for carbapenem-resistant *Klebsiella pneumoniae* infection: a systematic review and meta-analysis

**DOI:** 10.3389/fpubh.2026.1773088

**Published:** 2026-03-20

**Authors:** Zhenghaoyu Huang, Han Lin, Yonghong Guo

**Affiliations:** 1 Department of Medical Technology, School of Gongli Hospital Medical Technology, University of Shanghai for Science and Technology, Shanghai, China; 2 Department of Infectious Diseases, Gongli Hospital of Shanghai Pudong New Area, Shanghai, China

**Keywords:** carbapenem resistance, infection, *Klebsiella pneumoniae*, meta-analysis, risk factor

## Abstract

**Background:**

Carbapenem-resistant *Klebsiella pneumoniae* (CRKP) infections are increasingly prevalent. This meta-analysis aimed to identify key risk factors to inform prevention strategies.

**Methods:**

We searched PubMed and Web of Science up to October 2025 for studies on CRKP infection risk factors. Study quality was assessed using the Newcastle-Ottawa Scale. We computed pooled odds ratios (ORs) with 95% confidence intervals (CIs) and evaluated heterogeneity using the *I*^2^ statistic and the *Q*-test, employing either fixed- or random-effects models as appropriate.

**Results:**

Seventeen studies (5,303 cases) were included. Prior hospitalization, APACHE II score, and diabetes were not associated with CRKP infection. Significant risk factors included ICU admission (OR = 4.42, 95% CI: 2.82–6.91), cardiovascular disease (OR = 4.52, 95% CI: 3.23–6.30), renal disease (OR = 2.31, 95% CI: 1.74–3.06), multiple invasive procedures (e.g., mechanical ventilation OR = 3.82, 95% CI: 2.46–5.92; urinary catheterization OR = 4.73, 95% CI: 3.33–6.72), and exposure to various antimicrobial classes, notably carbapenems (OR = 6.36, 95% CI: 5.16–7.83), and tetracyclines (OR = 11.79, 95% CI: 7.75–17.93), though the latter’s high magnitude may be influenced by the limited number of studies and potential confounding by indication. Subgroup analysis showed the risks from mechanical ventilation and urinary catheterization were significantly higher in developing versus developed countries (*p* = 0.03 and *p* = 0.01, respectively).

**Conclusion:**

ICU admission, specific comorbidities, invasive procedures, and broad antimicrobial exposure are key risk factors for CRKP infection. Enhanced aseptic techniques in developing countries are crucial to mitigate risk.

**Systematic review registration:**

https://www.crd.york.ac.uk/PROSPERO/, CRD420251242025.

## 1 Introduction

Over the past decade, carbapenem-resistant Enterobacterales (CRE) have emerged as a major threat associated with antibiotic resistance. The US Centers for Disease Control and Prevention has designated them as “urgent pathogens” to human health (1). As a common cause of nosocomial infections, carbapenem-resistant *Klebsiella pneumoniae* (CRKP) accounts for 60–90% of clinical CRE infections and is widely prevalent in healthcare settings (2–4). CRKP infections are associated not only with high morbidity and mortality rates but also with prolonged hospital stays and substantial healthcare costs. Consequently, preventing and controlling CRKP infections has become one of the most challenging issues in clinical management.

With the rapid advancement of global healthcare, CRKP has emerged as an increasingly severe public health threat. Numerous studies indicate that CRKP infections are on the rise worldwide (5, 6). As listed in the World Health Organization (WHO) 2024 Priority Bacterial Pathogens List,^1^ CRKP has been designated as a pathogen of the highest priority, reflecting its significant threat to public health. In China, the resistance rate of *Klebsiella pneumoniae* has also surged rapidly. Data from the Chinese Antimicrobial Resistance Surveillance Network (CHINET, http://www.chinets.com) reveal that resistance rates of *Klebsiella pneumoniae* to imipenem and meropenem have skyrocketed from 3.0 and 2.9% in 2005 to 25.5 and 24.6% in 2025. To further curb the proliferation and spread of CRKP, scholars worldwide have conducted multiple retrospective studies on risk factors for CRKP infection, aiming to reduce the incidence risk at its source. However, existing studies often suffer from small sample sizes, incomplete coverage of risk factor indicators, and biases among study subjects. Therefore, this paper employs meta-analysis to systematically evaluate risk factors for CRKP infection, providing theoretical support for developing clinical treatment protocols and optimizing infection control measures.

## 2 Methods

This meta-analysis was performed and reported in accordance with the Preferred Reporting Items for Systematic Reviews and Meta-Analyses (PRISMA) guidelines (7). The protocol has been registered in the International Prospective Register of Systematic Reviews database (PROSPERO registration number: CRD420251242025).

### 2.1 Search strategy

Relevant studies published from inception up to 31 October 2025 for articles regarding risk factors for CRKP infection were searched in the PubMed and Web of Science (WOS). The search terms included a combination of the following terms: (i) *Klebsiella pneumoniae*; (ii) carbapenem-resistant or carbapenemases-producing or *Klebsiella pneumoniae* carbapenemases (KPC); (iii) infections; and (iv) risk factors. Reference lists cited by eligible retrieved articles were also manually retrieved to maximize inclusion of studies. Only full-text articles available in English were screened for inclusion.

### 2.2 Inclusion and exclusion criteria

The inclusion criteria were as follows: (i) study on risk factors for carbapenem-resistant infection, including the basic characteristics of the study and evaluation of methodological quality; (ii) case–control study; (iii) complete calculation of the odds ratio (OR) and 95% confidence interval (CI) provided in the literature; (iv) the case group was infected by CRKP and the control group was infected by carbapenem-sensitive *Klebsiella pneumoniae* (CSKP); (v) the study designs were comparable, specifically adhering to standard case–control methodologies with clearly defined criteria for the selection of cases (CRKP) and controls (CSKP); and (vi) baseline data for the case and control groups were comparable.

The exclusion criteria included: (i) reviews, systematic reviews, meta-analyses, editorials, letters to the editor, comments, and case reports; (ii) Incomplete information and data cannot provide or calculate complete OR values; (iii) studies that focused on neonatal and pediatric populations; (iv) the case group was not the CRKP infection group, with no control group and incomplete basic data; (v) duplicate reports.

### 2.3 Data extraction and quality assessment

Data were extracted from the included studies by two independent reviewers using the Microsoft Excel data abstraction form. Information regarding the general characteristics of the studies included the first author, country, study design, year of publication, risk factors for infections, number of patients with CRKP infections and number of patients with CSKP infections. Risk factor variables explored in fewer than two eligible studies were excluded. The Newcastle-Ottawa Quality Assessment Scale (NOS) was used for quality assessment. For each study that met the inclusion criteria, two researchers independently evaluated the quality based on three parts and eight clauses. The evaluation criteria were as follow: (i) study population selection: whether the case was appropriate or not; case representation; control selection; control determination; (ii) comparability between groups: comparability between case group and control group was considered in relation to statistical analysis; and (iii) measurement of exposure factors: determination of exposure factors; use of the same method to determine the exposure factors of the case and control groups; no response rate. There were 9 scores in quality evaluation using the NOS scale (≥7 for high-quality studies and <5 for low-quality studies).

### 2.4 Statistical analysis

Review Manager (version 5.4 software) and Stata (version 18.0 software) were used for statistical analysis. *Q*-test was used to test the heterogeneity of each study, and the size of heterogeneity was judged according to the heterogeneity evaluation index. Pooled odds ratios (ORs) and 95% CIs were calculated to express binary outcome results, while the mean difference (MD) and 95% CIs were used to express continuous outcome results. Sensitivity analysis on the literature included was done by omitting each study one by one at a time in the process of meta-analysis to inspect the change of merging effect, thus demonstrating the stability and accuracy of the outcome. Publication bias was also assessed using funnel plot and eggers test. Characteristics with fewer than 10 included studies were excluded from publication bias testing.

## 3 Results

### 3.1 Characteristics of included studies in systematic and meta-analysis

**Figure 1:**
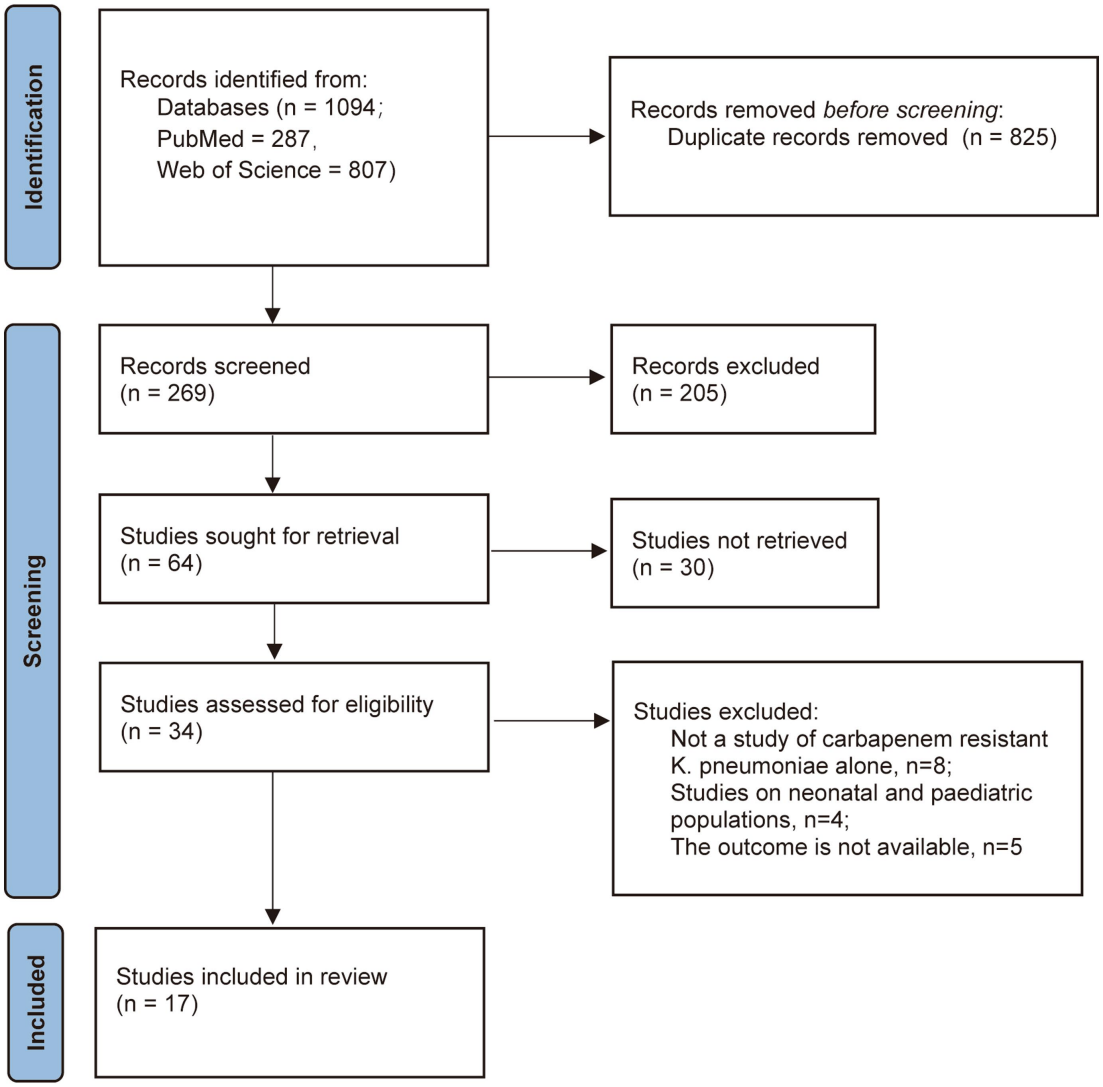
PRISMA flow chart.

A total of 1,094 studies were retrieved, of which 825 were excluded as they were not relevant to the topic and 235 were excluded as they met the exclusion criteria after reading the questions and abstracts. Further reading of the full-text of the remaining 34 articles (8–24) was conducted. According to the inclusion criteria and relevant indicators of the study, 17 articles were ultimately included in the meta-analysis (Figure 1). The publication date ranged from 2007 to 2025 and all were case–control studies. The CRKP infection group included 1907 cumulative cases and the control group included 3,396 cases. The basic characteristics of the studies are shown in Table 1. Based on the consistency of each findings, 25 risk factors including prior hospitalization, ICU admission, APACHE II score, diabetes mellitus, cardiovascular disease, renal disease, surgery, central venous catheterization, mechanical ventilation, urinary catheterization, tracheal cannula, tracheostomy, nasogastric catheterization, blood purification, carbapenems exposure, quinolones exposure, glycopeptides exposure, cephalosporins exposure, penicillins exposure, tetracyclines exposure, aminoglycosides exposure, polymyxins exposure, *β*-lactam/*β*-lactamase inhibitor combinations, corticosteroids and immunosuppression were analyzed.

**Table 1:** The baseline characteristics and quality of the included studies.

Author	Year	Country	Study design	CRKP (*n*)	CSKP (*n*)	Risk factors	NOS score
Lin, C. J (8)	2025	China	R, CCS	58	184	2,5,6,8,9,10,11,13,14,15	6
Guan, J (9)	2025	China	R, CCS	154	233	8,10,15,25	7
Zhou, X (10)	2024	China	R, CCS	74	78	1,3,8,9,10,13	6
Şimşek Bozok (11)	2025	Turkey	R, CCS	66	41	9,15	5
Cheng, Y (12)	2024	China	R, CCS	50	84	2,5,8,9,10,13,15,16,17,20	6
Çölkesen, F (13)	2023	Turkey	R, CCS	132	150	1,2,5,7,8,9,15,16,18	5
Lou, T (14)	2022	China	R, CCS	422	948	2,7,10,11,12,13,14,15,17,18,19,21,23,24	7
Li, Y (15)	2020	China	R, CCS	164	328	2,3,4,6,7,8,10,11,12,13,14,15,17,18,21	6
Cienfuegos (16)	2019	Colombia	R, CCS	49	289	2,8,9,10,15,16,18,22	6
Wang, Z (17)	2018	China	R, CCS	48	48	2,5,6,9,10,13,14,15,16,18,20	6
Falagas, M (18)	2007	Greece	R, CCS	53	53	2,9,12,15,17,18,19	7
Alparslan, V (19)	2025	Turkey	R, CCS	159	130	3,4,5,8,17,25	6
Gupta, P (20)	2021	India	R, CCS	85	26	2,10,15,23	5
Xiao, T (21)	2020	China	R, CCS	104	267	1,2,3,8,9,10,13,14,15,16,18,20,21,22,24	7
Madueño, A (22)	2017	Spain	R, CCS	87	200	1,4,6,8,9,10,15,16,18,19,24,25	5
Mills, J. P (23)	2016	America	R, CCS	99	123	9,12,15,16,17,24,25	6
Hussein, K (24)	2013	Israel	R, CCS	103	214	6,8,9,14,15,16,17,18,19,22,23	6

R, retrospective; CCS, case–control study; CRKP, carbapenem-resistant *Klebsiella pneumoniae*; CSKP, carbapenem-susceptible *Klebsiella pneumoniae*; NOS, Newcastle-Ottawa Scale; Risk factors: 1. Prior hospitalization; 2. ICU admission; 3. APACHE II score; 4. Diabetes mellitus; 5. Cardiovascular disease; 6. Renal disease; 7. Surgery; 8. Central venous catheterization; 9. Mechanical ventilation; 10. Urinary catheterization; 11. Tracheal cannula; 12. Tracheostomy; 13. Nasogastric catheterization; 14. Blood purification; 15. Carbapenems; 16. Cephalosporins; 17. Glycopeptides; 18. Quinolones; 19. Penicillins; 20. *β*-lactam/*β*-lactamase inhibitor combinations; 21. Tetracyclines; 22. Aminoglycosides; 23. Polymyxins; 24. Corticosteroids; 25. Immunosuppression.

### 3.2 Risk factor analysis

**Figure 2:**
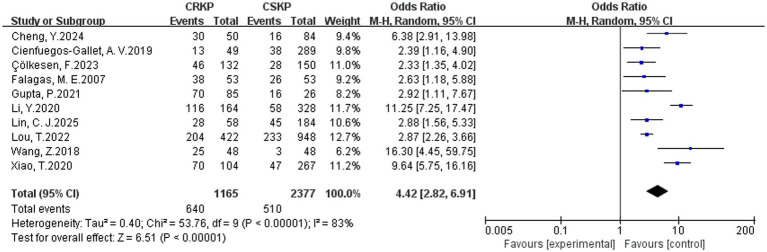
Forest plot of ICU admission.

**Figure 3:**
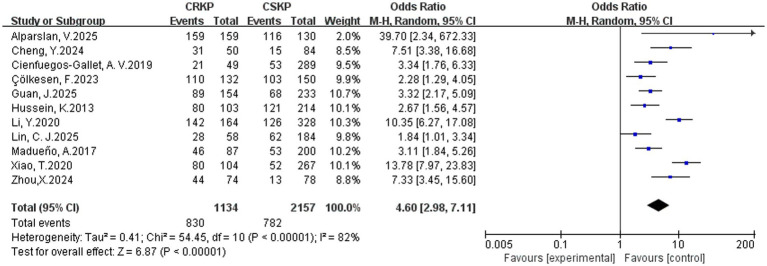
Forest plot of central venous catheterizations.

**Figure 4:**
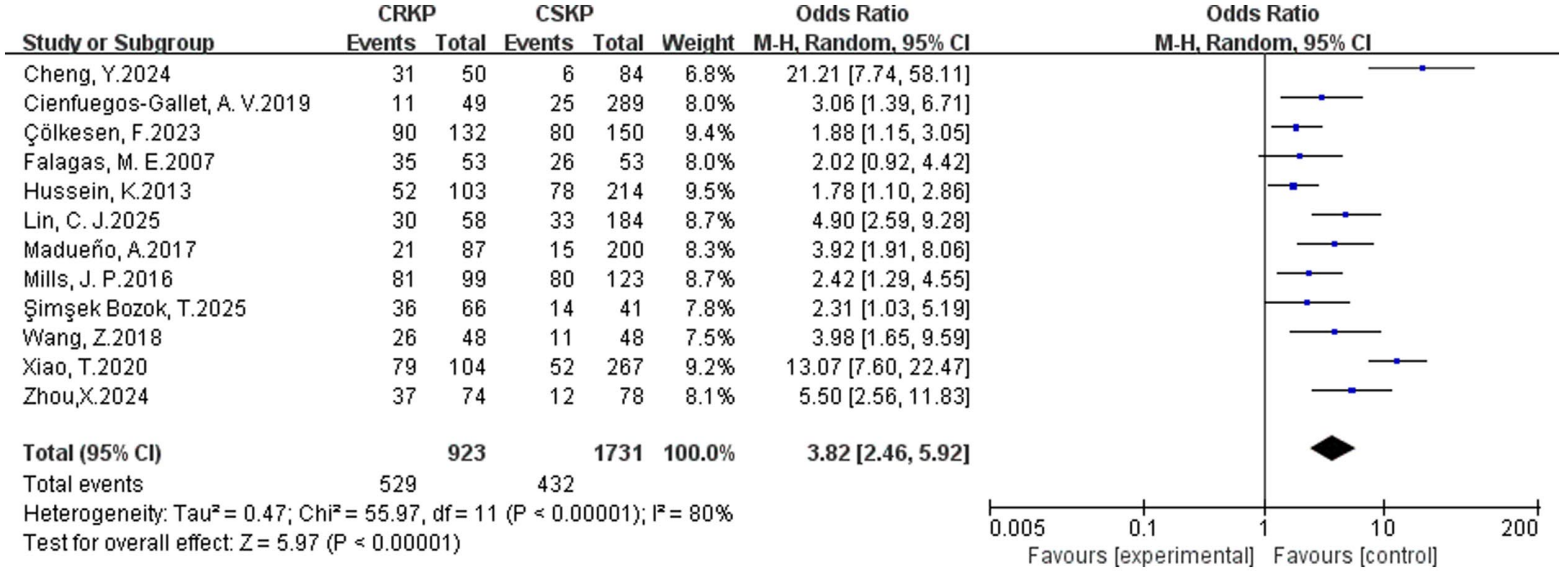
Forest plot of mechanical ventilation.

**Figure 5:**
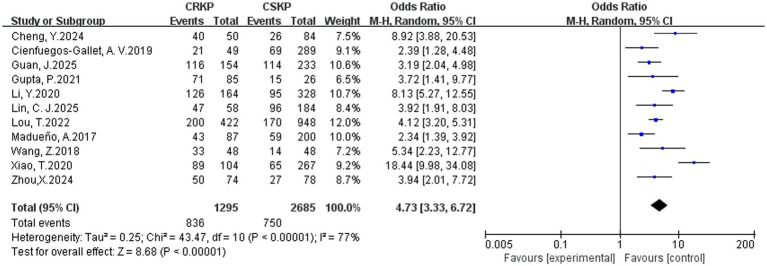
Forest plot of urinary catheterization.

**Figure 6:**
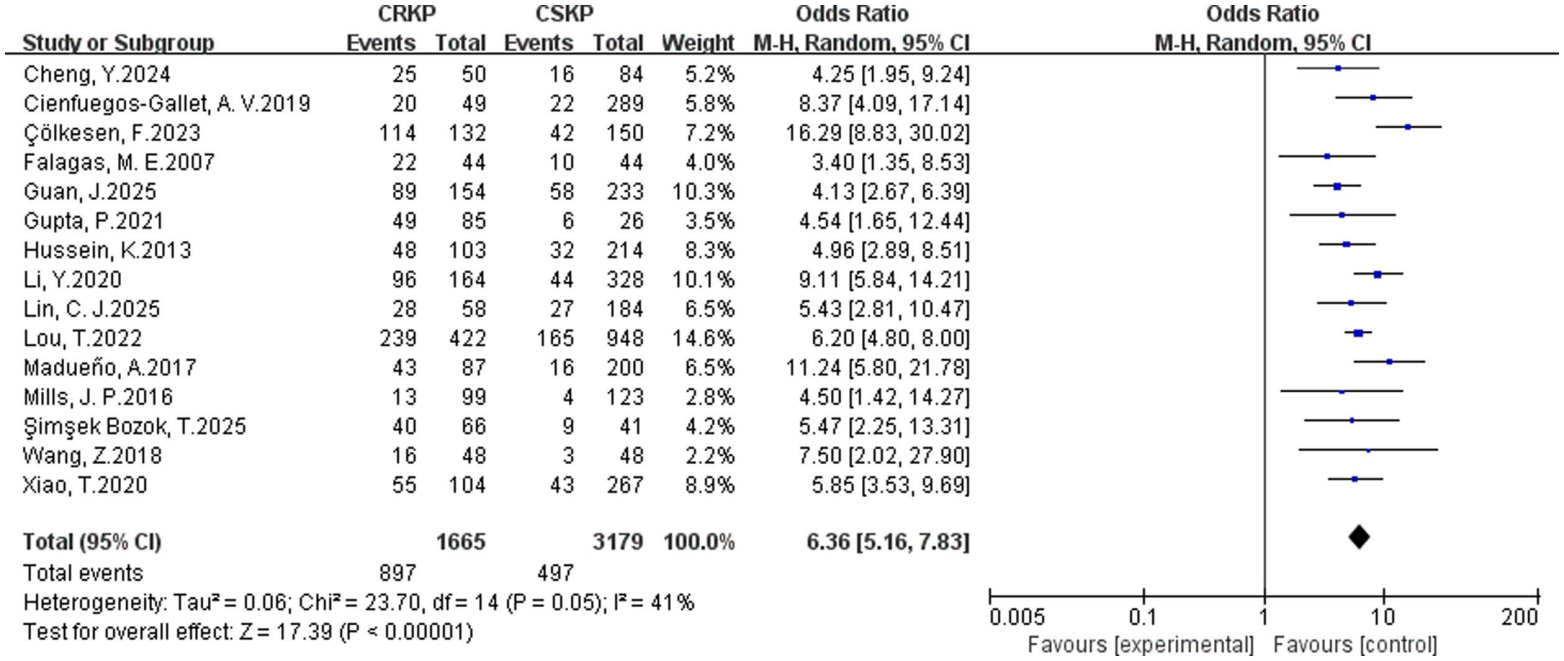
Forest plot of carbapenems exposure.

In terms of the analysis on heterogeneity of risk factors, there was no significant statistical heterogeneity regarding cardiovascular disease, renal disease, surgery, tracheostomy, tracheal cannula, glycopeptides, *β*-lactam/*β*-lactamase inhibitor combinations, tetracyclines, Polymyxins, aminoglycosides, corticosteroids and immunosuppression, whereas other factors demonstrated heterogeneity. Results of the fixed-effects analysis model showed that cardiovascular disease (OR = 4.52, 95% CI: 3.23–6.30), renal disease (OR = 2.31, 95% CI: 1.74–3.06), surgery (OR = 2.87, 95% CI: 2.33–3.53), tracheal cannula (OR = 5.13, 95% CI: 3.65–7.20), tracheostomy (OR = 4.25, 95% CI: 3.07–5.90), glycopeptides exposure (OR = 4.68, 95% CI: 3.77–5.81), *β*-lactam/*β*-lactamase inhibitor combinations (OR = 3.38, 95% CI: 2.36–4.83), tetracyclines exposure (OR = 11.79, 95% CI: 7.75–17.93), aminoglycosides exposure (OR = 4.28, 95% CI: 2.47–7.42), polymyxins exposure (OR = 10.39, 95% CI: 4.51–23.91), corticosteroids use (OR = 2.81, 95% CI: 2.08–3.78) and immunosuppression (OR = 3.00, 95% CI: 2.09–4.29) significantly increased the risk of CRKP infection. No statistically significant associations were found for prior hospitalization, APACHE II score and diabetes mellitus with CRKP infection. Results of the random-effects analysis model showed that ICU admission (OR = 4.42, 95% CI: 2.82–6.91; Figure 2), Central venous catheterization (OR = 4.60, 95% CI: 2.98–7.11; Figure 3), Mechanical ventilation (OR = 3.82, 95% CI: 2.46–5.92; Figure 4), Urinary catheterization (OR = 4.73, 95% CI: 3.33–6.72; Figure 5), Nasogastric catheterization (OR = 6.16, 95% CI: 3.31–11.49), Blood purification (OR = 4.78, 95% CI: 2.98–7.67), Carbapenems exposure (OR = 6.36, 95% CI: 5.16–7.83; Figure 6), Cephalosporins exposure (OR = 6.21, 95% CI: 3.82–10.10), Quinolones exposure (OR = 4.65, 95% CI: 2.58–8.39) and Penicillins exposure (OR = 3.05, 95% CI: 1.51–6.17) significantly increased the risk of CRKP infection. However, it is essential to provide context for the extraordinarily high ORs observed for tetracyclines (OR = 11.79, 95% CI: 7.75–17.93) and polymyxins (OR = 10.39, 95% CI: 4.51–23.91). These extreme values should be interpreted with caution, as they are likely influenced by confounding by indication—these agents are typically reserved as last-resort treatments for patients already suffering from highly complex or refractory infections. The details are shown in Table 2.

**Table 2:** Results of a meta-analysis on risk factors for CRKP infection.

Risk factors	Number of studies	*I*^2^ (%)	*p* value of heterogeneity	Model	Pooled OR/MD (95%CI)	*p* value	Egger test
Characteristics							
Prior hospitalization	4	88	<0.001	RE	2.19 (0.86, 5.59)	0.10	–
ICU admission	10	83	<0.001	RE	4.42 (2.82, 6.91)	<0.001	0.493
APACHE II score	4	95	<0.001	RE	3.34 (−0.11, 6.78)	0.06	–
Comorbidities							
Diabetes mellitus	3	93	<0.001	RE	1.16 (0.41, 3.32)	0.78	–
Cardiovascular disease	5	34	0.19	FE	4.52 (3.23, 6.30)	<0.001	–
Renal disease	5	0	0.56	FE	2.31 (1.74, 3.06)	<0.001	–
Invasive procedures							
Surgery	3	0	0.50	FE	2.87 (2.33, 3.53)	<0.001	–
Central venous catheterization	11	82	<0.001	RE	4.60 (2.98, 7.11)	<0.001	0.480
Mechanical ventilation	12	80	<0.001	RE	3.82 (2.46, 5.92)	<0.001	0.285
Urinary catheterization	11	77	<0.001	RE	4.73 (3.33, 6.72)	<0.001	0.686
Tracheal cannula	3	0	0.45	FE	5.13 (3.65, 7.20)	<0.001	–
Tracheostomy	4	36	0.20	FE	4.25 (3.07, 5.90)	<0.001	–
Nasogastric catheterization	7	86	<0.001	RE	6.16 (3.31, 11.49)	<0.001	–
Blood purification	6	58	0.03	RE	4.78 (2.98, 7.67)	<0.001	–
Antimicrobial exposure							
Carbapenems	15	41	0.05	RE	6.36 (5.16, 7.83)	<0.001	0.877
Cephalosporins	8	68	0.002	RE	6.21 (3.82, 10.10)	<0.001	–
Glycopeptides	7	9	0.36	FE	4.68 (3.77, 5.81)	<0.001	–
Quinolones	9	89	<0.001	RE	4.65 (2.58, 8.39)	<0.001	–
Penicillins	4	86	<0.001	RE	3.05 (1.51, 6.17)	0.002	–
*β*-lactam/*β*-lactamase inhibitor combinations	3	0	0.72	FE	3.38 (2.36, 4.83)	<0.001	–
Tetracyclines	3	0	0.79	FE	11.79 (7.75, 17.93)	<0.001	–
Aminoglycosides	3	47	0.15	FE	4.28 (2.47, 7.42)	<0.001	–
Polymyxins	3	0	0.84	FE	10.39 (4.51, 23.91)	<0.001	–
Corticosteroids	4	17	0.31	FE	2.81 (2.08, 3.78)	<0.001	–
Immunosuppression	4	0	0.85	FE	3.00 (2.09, 4.29)	<0.001	–

CI, confidence interval; CRKP, carbapenem-resistant *Klebsiella pneumoniae*; OR, odds ratio; MD, mean difference; RE, random effects model; FE, fixed effects model. “–,” represents less than 10 articles.

### 3.3 Subgroup analysis

Next, subgroup analysis will be conducted based on countries with different levels of economic development. Five characteristics were included in the study: carbapenems, ICU admission, central venous catheterization, mechanical ventilation, and urinary catheterization. Among these, the risk of CRKP infection differed significantly between the two subgroups in terms of both mechanical ventilation (*p* = 0.03) and urinary catheterization (*p* = 0.01). No statistically significant associations were found for carbapenems, ICU admission and central venous catheterization with CRKP infection. The details are shown in Table 3.

**Table 3:** Subgroup analysis for of CRKP infection risk in different countries.

Variable	Developing countries	No. of studies	*I*^2^ (%)	OR (95%CI)	Developed countries	No. of studies	*I*^2^ (%)	OR (95%CI)	*p* value
Carbapenems		11	44	6.45 (5.52, 7.53)		4	47	5.86 (4.09, 8.39)	0.68
ICU admission		9	85	4.66 (2.88, 7.55)		1	-	2.63 (1.18, 5.88)	0.23
Central venous catheterization		9	83	5.22 (3.08, 8.86)		2	0	2.89 (1.98, 4.21)	0.07
Mechanical ventilation		8	83	4.90 (2.70, 8.89)		4	11	2.28 (1.64, 3.17)	**0.03** ^*^
Urinary catheterization		10	75	5.12 (3.57, 7.35)		1	–	2.34 (1.39, 3.92)	**0.01** ^*^

* indicates that the *p*-value <0.05 is statistically significant.

### 3.4 Sensitivity analysis

In this research, the sensitivity analysis was performed through eliminating each included study one by one. It found that the combined OR value, 95%CI and *p* value after omission were very close in most of the risk factors to the results when the study was not omitted. Nevertheless, when the study of Madueño (22) was removed, the ORs and the corresponding 95%CI for prior hospitalization changed from 2.19 (95%CI: 0.86–5.59) to 3.45 (95%CI: 1.71–6.98). When the study of Li et al. (15) was removed, the ORs and the corresponding 95% CI for diabetes mellitus changed from 1.16 (95%CI: 0.41–3.32) to 1.93 (95%CI: 1.33–2.80). The results and statistically significant difference changed for the prior hospitalization factor and diabetes mellitus factor. This finding suggests that the pooled estimates for prior hospitalization and diabetes mellitus are highly sensitive to the influence of individual studies and therefore lack statistical robustness. The direction and magnitude of association for these two variables cannot be considered stable based on the current evidence.

### 3.5 Publication bias

**Figure 7:**
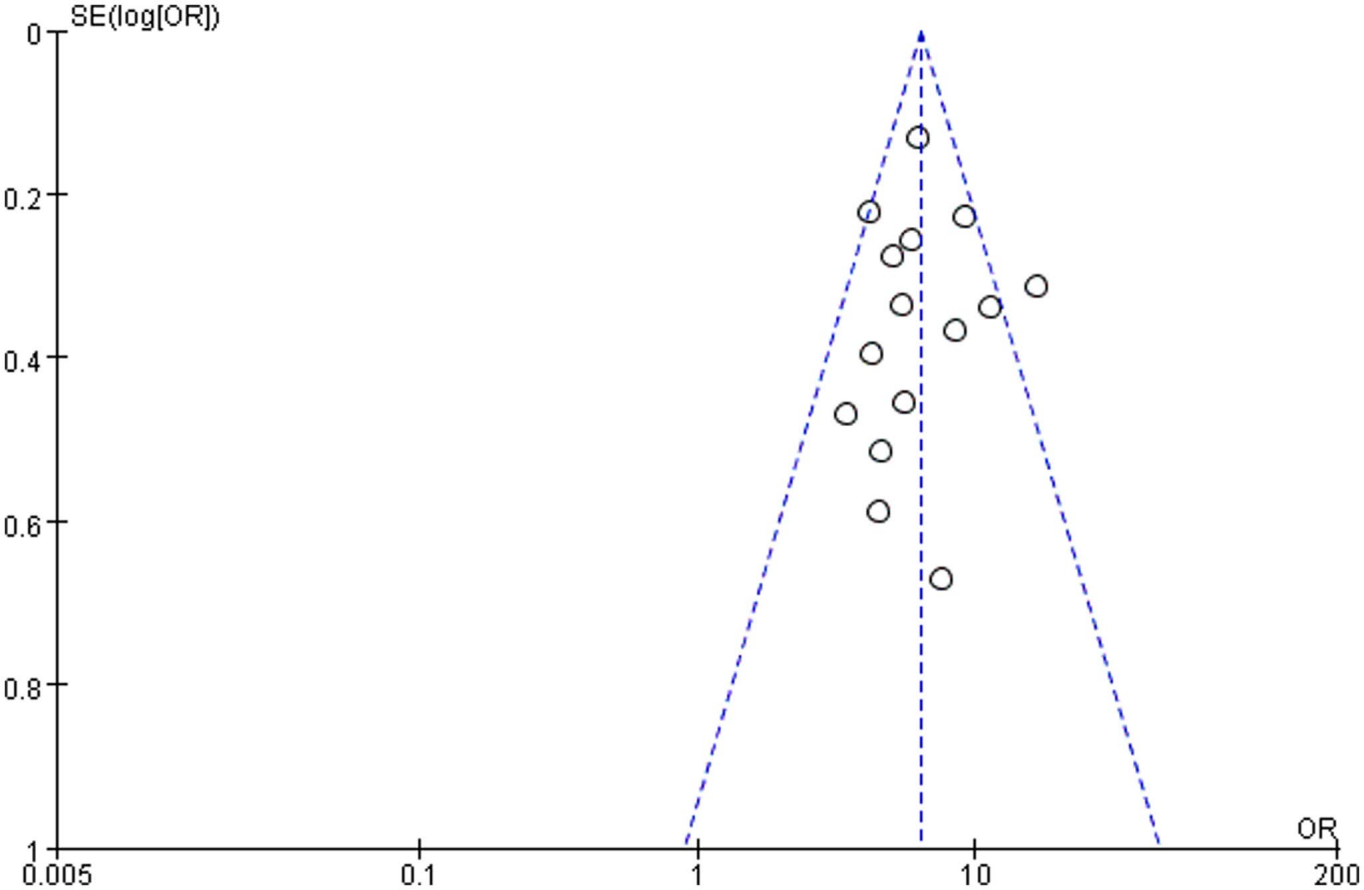
Funnel plot to assess publication bias for carbapenems exposure.

In this study, publication bias for each related factor was identified by funnel plot and Egger regression. No distinct asymmetry was found in each funnel plot indicating that the publication bias was basically balanced. The most representative funnel plot is shown in Figure 7. Egger regression test results showed no significant publication bias in studies reporting the ICU admission (Egger: 0.493), central venous catheterization (Egger: 0.480), mechanical ventilation (Egger: 0.285), the urinary catheterization (Egger: 0.686) and carbapenems (Egger: 0.877). The details are shown in Table 2. Risk factors with fewer than 10 studies included were not subjected to funnel plot analysis or Egger’s regression test.

## 4 Discussion

Since its first discovery in the United States in 1996, carbapenem-resistant *Klebsiella pneumoniae* (CRKP) has become the most common carbapenem-resistant Enterobacteriaceae species in the United States (2). This strain has also become endemic in China (25), some South American countries (26), and Europe (3). According to epidemiological data from monitoring systems, the proportion of patients infected with CRKP in China (49%) is much higher than in the United States (25.9%) and South America (21.7%) (26). CRKP is rapidly spreading worldwide and has drawn significant attention. In recent years, with the continuous increase in the infection rate of CRKP, the mortality rate of patients has also risen. Infections caused by such strains are difficult to eradicate and have limited treatment options, which greatly increases the cost of medical public health. Therefore, it is particularly important to identify the risk factors for CRKP infection. By screening high-risk populations, clinicians can take effective preventive measures early, optimize the selection and efficacy evaluation of empirical treatment regimens, and thus save medical resources. This meta-analysis aims to systematically review the risk factors for CRKP infection and analyze the differences in CRKP infection risk among countries at different levels of economic development.

This analysis included 17 original studies on the epidemiological data of CRKP infection. The review evaluated the risk factors for CRKP infection in 5,303 patients from eight countries between 2007 and 2025, and the results showed that there were differences in the risk of CRKP infection among countries with different levels of economic development. In addition, due to the inconsistent conclusions of previous studies on the risk factors related to CRKP infection, a systematic summary of the existing evidence is crucial to assist clinicians in the early diagnosis and management of high-risk patients. This meta-analysis revealed that ICU admission, comorbidities, invasive procedures, and antimicrobial exposure were risk factors for CRKP infection in patients.

For the analysis of ICU admission, a total of 10 studies comprising 3,542 cases were included. The OR was 4.42 (95%CI: 2.82–6.91) based on the random-effects model, indicating that ICU admission was a risk factor for CRKP infection. CRKP, as one of the important pathogens causing hospital-acquired infections, is highly resistant to drugs and is commonly found in patients who have been hospitalized for a long time and those with weakened immune systems. ICU patients, who often have severe underlying diseases, compromised immune functions and weak self-defense barriers, are highly susceptible to CRKP colonization and infection. At the same time, the frequent movement of people (doctors, nurses, healthcare attendants, and family members) in the ICU and the surfaces of medical devices such as ventilators, monitors, and bed units can easily become “reservoirs” of CRKP. If the disinfection process is not standardized, cross-infection is highly likely to occur. Moreover, another study also indicated that contaminated handwashing sinks within ICUs are a significant source of CRKP transmission (27). This highlights the need for healthcare workers to enhance their awareness of disinfection protocols and adhere to standardized procedures to mitigate the risk of CRKP spread.

For the analysis of comorbid conditions, cardiovascular disease and renal disease were evaluated separately, with 5 studies included for each condition, involving 1,043 and 1,434 cases, respectively. The odds ratios based on the fixed-effect model were 4.52 (95%CI: 3.23–6.30) and 2.31 (95%CI: 1.74–3.06), indicating that cardiovascular disease and renal disease are risk factors for CRKP infection. Patients with cardiovascular disease face the highest risk (OR = 4.52, 95% CI: 3.23–6.30), potentially due to the prevalence of immune dysregulation, frequent invasive procedures, and prolonged hospitalization in this population. Patients with renal disease, particularly those requiring dialysis, face unique risks due to vascular access and uremia-related immunosuppression. This underscores the need for more aggressive risk stratification and targeted prevention strategies in hospitalized patients with these comorbidities. Such measures should include proactive culture monitoring, enhanced contact isolation, and optimized antibiotic use to interrupt CRKP transmission among this most vulnerable population.

Notably, this meta-analysis did not identify prior hospitalization, APACHE II score, or diabetes mellitus as significant risk factors for CRKP infection, which appears inconsistent with findings reported in several previous studies. Several potential explanations may account for this discrepancy. First, differences in study populations may have influenced the pooled results. The included studies varied substantially in patient characteristics, clinical settings (e.g., ICU vs. general wards), and geographic regions, which could modify the strength or direction of these associations. Second, the definitions of exposure were not uniform across studies. In particular, the timeframe used to define “prior hospitalization” differed markedly (ranging from recent hospitalization within 30 days to any hospitalization within the previous year), potentially diluting its overall effect when combined in a meta-analysis. Third, adjustment for confounding factors varied across studies. Some studies reported unadjusted estimates, while others controlled for multiple clinical variables, which may have led to inconsistent effect estimates for APACHE II score and diabetes mellitus. Finally, the specific context of the included studies should be considered. In settings with a high endemic burden of CRKP, factors such as antimicrobial exposure and invasive procedures may play a more dominant role, thereby attenuating the independent effects of baseline severity scores or chronic comorbidities. Therefore, these findings should be interpreted cautiously, and future well-designed studies with standardized exposure definitions and rigorous confounder adjustment are needed to further clarify the roles of these factors in CRKP infection risk.

Analysis of invasive procedures revealed that all procedures were strong risk factors for CRKP infection, such as surgery (OR = 2.87, 95% CI: 2.33–3.53), mechanical ventilation (OR = 3.82, 95% CI: 2.46–5.92), urinary catheterization (OR = 4.73, 95% CI: 3.33–6.72), nasogastric catheterization (OR = 6.16, 95% CI: 3.31–11.49), and blood purification (OR = 4.78, 95% CI: 2.98–7.67). It has been well documented that infection or colonization by resistant pathogens is associated with the use of invasive procedures (28). Prior surgery is a recognized risk factor facilitating bacterial translocation and subsequent infection (29). Urinary catheterization can compromise the urethral mucosal barrier and facilitate biofilm formation, leading to persistent CRKP colonization (30). Mechanical ventilation increases the risk of CRKP aspiration due to respiratory muscle weakness and tracheal mucosal injury, thereby inducing infection (31). Furthermore, *Klebsiella pneumoniae* has demonstrated a significant ability to colonize medical devices and equipment, enabling transmission between patients (32). These invasive procedures increase the risk of CRKP infection. Clinicians should enhance device disinfection, conduct regular monitoring of airway colonization, and evaluate the necessity of early extubation to reduce unnecessary invasive procedures and lower the likelihood of CRKP infection. Subgroup analysis revealed significant differences between developing and developed countries in the risk of CRKP infections associated with mechanical ventilation (OR = 4.90 vs. OR = 2.28) and urinary catheterization (OR = 5.12 vs. OR = 2.34), with these differences being statistically significant (*p* = 0.03, *p* = 0.01). This disparity underscores that in regions with relatively limited healthcare resources, the quality of infection control measures implemented during invasive procedures may be a more critical risk determinant than the procedures themselves. Therefore, infection control efforts in developing countries must prioritize enhancing sterile technique standards for relevant procedures, improving equipment sterilization levels, and boosting hand hygiene compliance to effectively reduce preventable infection risks. However, the results of subgroup analyses should be interpreted with caution. For several variables, including ICU admission and urinary catheterization, the subgroup of developed countries was represented by only one eligible study, resulting in limited statistical power and reduced robustness of the pooled estimates. The highly uneven distribution of studies between developing and developed countries may have constrained the reliability of between-group comparisons. Future high-quality, multicenter studies from underrepresented settings, particularly developed countries, are needed to validate these subgroup findings and enhance their generalizability.

Analysis of antimicrobial exposure revealed that antimicrobial exposure is the most critical risk factor for CRKP infection. This study confirmed that carbapenems exposure (OR = 6.36, 95% CI: 5.16–7.83), cephalosporins exposure (OR = 6.21, 95% CI: 3.82–10.10), glycopeptides exposure (OR = 4.68, 95% CI: 3.77–5.81), quinolone exposure (OR = 4.65, 95% CI: 2.58–8.39), penicillins exposure (OR = 3.05, 95% CI: 1.51–6.17), tetracyclines exposure (OR = 11.79, 95% CI: 7.75–17.93), aminoglycosides exposure (OR = 4.28, 95% CI: 2.47–7.42), and polymyxins exposure (OR = 10.39, 95% CI: 4.51–23.91) were all significantly associated with an increased risk of CRKP infection.

One of the primary factors contributing to infections caused by drug-resistant bacteria is the selective pressure exerted by antibiotics. Long-term use of carbapenem antibiotics and combination therapy exerts strong selective pressure on the gut microbiota, disrupting the human microbiome balance. This suppresses or eradicates large numbers of carbapenem-susceptible *Klebsiella pneumoniae* (CSKP), thereby creating conditions for the colonization and dominant proliferation of carbapenem-resistant *Klebsiella pneumoniae* (CRKP). Research by Kuang et al. (33) indicates that even low-dose carbapenem use can lead to CRKP colonization in the gut, suggesting that clinicians should exercise caution when prescribing antimicrobial agents. Additionally, carbapenem resistance can be disseminated through resistance plasmids, contributing to the spread of infections caused by carbapenem-resistant *Klebsiella pneumoniae* (CRKP) (34). More critically, the production of *Klebsiella pneumoniae* carbapenemase (KPC) constitutes a key mechanism underlying carbapenem resistance. Prolonged use of carbapenems may promote the acquisition of KPC enzymes, which efficiently hydrolyze penicillins, cephalosporins, and carbapenems, thereby reducing their antimicrobial efficacy.

In clinical decision-making for CRKP infections, the role of cephalosporin antibiotics presents significant contradictions. On one hand, monotherapy with traditional cephalosporins is typically ineffective, failing not only to control infections but also to potentially increasing patient mortality risks by delaying effective treatment. The study has confirmed that inappropriate initial antibiotic selection is a key factor contributing to poor outcomes (6). On the other hand, novel “cephalosporin/beta-lactamase inhibitor” combination therapies, exemplified by ceftazidime-avibactam, offer a critical alternative for treating CRKP infections driven by specific resistance mechanisms such as KPC enzyme production. These agents particularly compensate for limitations of traditional drugs in multidrug-resistant settings (35).

We also found glycopeptides and quinolones were risk factors for CRKP infections, consistent with previous findings (36). Long-term use of glycopeptides significantly suppresses the growth of Gram-positive bacteria, potentially leading to increased mutation rates and drug resistance in Gram-negative bacteria and thereby elevating the risk of CRKP infections. But this hypothesis requires further validation through additional research. Moreover, multiple studies have indicated that clinical isolates of CRKP exhibit significant resistance to quinolones, consistent with the findings of this study (37, 38). A previous study conducted an epidemiological investigation of quinolone resistance determinants, including plasmid-mediated quinolone resistance (PMQR) genes and spontaneous mutations in the quinolone resistance-determining regions (QRDRs) of the gyrA and parC genes (39). Results indicated that mutations in the QRDRs of gyrA and parC are key factors contributing to the high prevalence of quinolone resistance in CRKP. Therefore, rational antibiotic use, particularly with quinolones, is essential when treating clinical CRKP infections.

A finding requiring particular attention is the significantly high-risk association between the use of tetracyclines (especially tigecycline) and polymyxins and CRKP infections. This does not imply that these drugs cause CRKP infections, but rather that they are often employed as the last line of defense against multidrug-resistant Gram-negative bacteria, including CRKP. Therefore, their use itself signifies that patients are already in a state of highly complex and refractory infection. When patients have used tetracyclines and polymyxins prior to infection, it strongly suggests that they may have had undiagnosed CRKP colonization or a long and complicated history of antimicrobial therapy. This underscores the imperative for clinicians to rigorously control indications for last-resort antibiotics like tigecycline, avoiding inappropriate or excessive empirical use to prevent further selection of more resistant strains. Concurrently, patients who have received such agents warrant heightened vigilance regarding subsequent risks of CRKP infection or colonization.

A critical methodological concern in this meta-analysis is the presence of extreme heterogeneity (*I*^2^ > 80%) for multiple factors, including nasogastric catheterization, ICU admission, central venous catheterization, and mechanical ventilation. These factors are closely linked to disease severity and underlying comorbidities, which may differ substantially across study populations. Moreover, variations in healthcare resource availability, infection prevention protocols, and antimicrobial stewardship programs across regions may have contributed to between-study variability. Although random-effects models were applied to account for statistical heterogeneity, such models cannot fully resolve underlying clinical heterogeneity. Consequently, the pooled odds ratios for these factors should be considered as indicative associations rather than precise quantitative risk estimates. Future studies with standardized exposure definitions and severity adjustment are required to reduce heterogeneity and enhance comparability.

## 5 Limitation

This meta-analysis has several limitations. First, the literature search was restricted to two major databases (PubMed and Web of Science) and to studies published in English. This restriction may have introduced selection bias by excluding relevant studies indexed in other databases such as Embase or the Cochrane Library, as well as studies published in non-English languages. Some regional evidence or unpublished data might not have been captured, which could have influenced the pooled effect estimates. Second, all the studies included were case–control investigations, yielding low-quality evidence for causation, and their methodological quality requires improvement. The overall methodological quality of the included studies was moderate, with 88% scoring≤6 on the NOS. While sensitivity analyses supported the stability of our findings, this inherent limitation highlights the need for more high-quality, large-scale studies to confirm these risk associations. In addition, for factors with fewer included studies, inadequate assessment of publication bias may have influenced the estimated results. Third, subgroup analyses were constrained by relatively limited data from developed countries, weakening the statistical power of relevant comparisons. Consequently, more high-quality primary studies from underrepresented settings are needed to validate these findings. Moreover, patients may exhibit complex underlying conditions, and no detailed stratification of disease characteristics was performed.

## 6 Conclusion

In this meta-analysis, ICU admission, underlying cardiovascular or renal disease, invasive procedures (such as central venous catheterization, mechanical ventilation, catheterization, etc.), and antimicrobial exposure (including carbapenems, cephalosporins, fluoroquinolones, tetracyclines, and polymyxins, etc.) were identified as risk factors for CRKP infection. To reduce the risk of CRKP infection, the use of invasive medical devices should be minimized whenever possible, and antibiotic use should be standardized to ensure rational prescribing practices, particularly in developing countries.

## Data Availability

The original contributions presented in the study are included in the article/Supplementary material, further inquiries can be directed to the corresponding author.

## Footnotes

1
www.who.int/publications/i/item/9789240093461
